# Intravitreal injection of anti-miRs against miR-142-3p reduces angiogenesis and microglia activation in a mouse model of laser-induced choroidal neovascularization

**DOI:** 10.18632/aging.203035

**Published:** 2021-05-05

**Authors:** Quentin Roblain, Thomas Louis, Cassandre Yip, Louis Baudin, Ingrid Struman, Vincenza Caolo, Vincent Lambert, Julie Lecomte, Agnès Noël, Stephane Heymans

**Affiliations:** 1 Laboratory of Tumor and Development Biology, GIGA-Cancer, University of Liège, Liège, Belgium; 2 Department of Cardiology, CARIM School for Cardiovascular Diseases, Faculty of Health, Medicine and Life Sciences, Maastricht University, Maastricht, The Netherlands; 3 Molecular Angiogenesis Laboratory, GIGA-Cancer, University of Liège, Liège, Belgium; 4 Ophthalmic Tissue Bank, Department of Ophthalmology, University Hospital of Liège, Sart-Tilman, Belgium; 5 Department of Cardiovascular Sciences, Centre for Molecular and Vascular Biology, KU Leuven, Leuven, Belgium

**Keywords:** miR-142-3p, age-related macular degeneration, angiogenesis, inflammation, microglia

## Abstract

Age-related macular degeneration (AMD) is a worldwide leading cause of blindness affecting individuals over 50 years old. The most aggressive form, wet AMD, is characterized by choroidal neovascularization (CNV) and inflammation involving microglia recruitment. By using a laser-induced CNV mouse model, we provide evidence for a key role played by miR-142-3p during CNV formation. MiR-142-3p was overexpressed in murine CNV lesions and its pharmacological inhibition decreased vascular and microglia densities by 46% and 30%, respectively. Consistently, miR-142-3p overexpression with mimics resulted in an increase of 136% and 126% of blood vessels and microglia recruitment. Interestingly, miR-142-3p expression was linked to the activation state of mouse microglia cells as determined by morphological analysis (cell solidity) through a computational method. *In vitro*, miR-142-3p overexpression in human microglia cells (HMC3) modulated microglia activation, as shown by CD68 levels. Interestingly, miR142-3p modulation also regulated the production of VEGF-A, the main pro-angiogenic factor. Together, these data strongly support the unprecedented importance of miR-142-3p-dependent vascular-inflammation axis during CNV progression, through microglia activation.

## INTRODUCTION

Globally, 2.2 billion people suffer of vision impairment or blindness [1]. Age-related macular degeneration (AMD) is the fourth leading cause of blindness worldwide [2], affecting 8.7 % of population. This percentage is expected to increase due to the ageing population [3]. Although less frequent than atrophic (or dry) AMD, neovascular (or wet) AMD represents the most vision-threatening form of the disease [4]. It is mainly characterized by choroidal neovascularization (CNV), the formation of abnormal blood vessels arising from the choroid and invading the subretinal space, leading to vision impairment [5]. This angiogenic process is associated with the recruitment and proliferation of inflammatory cells including neutrophils, macrophages and microglia [6, 7]. The latter cells are the main resident immune cells and contributors to the innate immunity within the central nervous system, including the retina. At the experimental level, rodent models of laser-induced CNV are the most broadly used for neovascular AMD pre-clinical research [7, 8]. They recapitulate the angiogenic and inflammatory phases of the disease including microglia implication. Antibody-based anti-VEGF (Vascular Endothelium Growth Factor) therapy is still the golden standard for the management of neovascular AMD [9]. Unfortunately, around one fifth of neovascular AMD patients do not or poorly respond to anti-VEGF therapy [10]. Therefore, additional therapies are urgently required.

MicroRNAs, also called miRs or miRNAs, are short non-coding RNA species of about 20-24 nucleotides length, which act as post-transcriptional gene-expression regulators. A single mature microRNA can bind to several mRNAs and a single mRNA can be targeted by several miRNAs [11–13]. Many ocular diseases are regulated by short non-coding RNAs, and a growing body of literature related to miRNA complications in eye disorders is currently emerging [14, 15]. To date, several teams have focused on establishing microRNAs as potential AMD biomarkers in both human and mouse study [16, 17]. However, only a few of these studies addressed the question of a functional role of microRNAs in AMD [18]. Through their capacity to regulate biological processes such as angiogenesis and inflammation [19, 20], a functional contribution of miRNA in AMD is expected, but poorly documented. Hence, miRNA as therapeutic target for the treatment of AMD is an emerging concept for future drug design.

In the present study, we searched for microRNAs with functional implication in CNV development. We first determined the expression profile of a set of angiogenesis and inflammation-related microRNAs in mouse CNV lesions. Inhibition and overexpression of miR-142-3p, one of the top dysregulated microRNAs, regulated both vascular and inflammatory phenotypes. Notably, microglia recruitment and activation were enhanced by miR-142-3p mimic and reduced by miR-142-3p inhibitor both *in vitro* and *in vivo*. Our work provides evidence that miR-142-3p is a functional mediator during CNV progression and acts as a microglia cell activator. MiR-142-3p is worth considering as a target for future neovascular AMD therapeutics.

## RESULTS

### miR-142-3p is overexpressed in a laser-induced CNV mouse model

**Figure 1:**
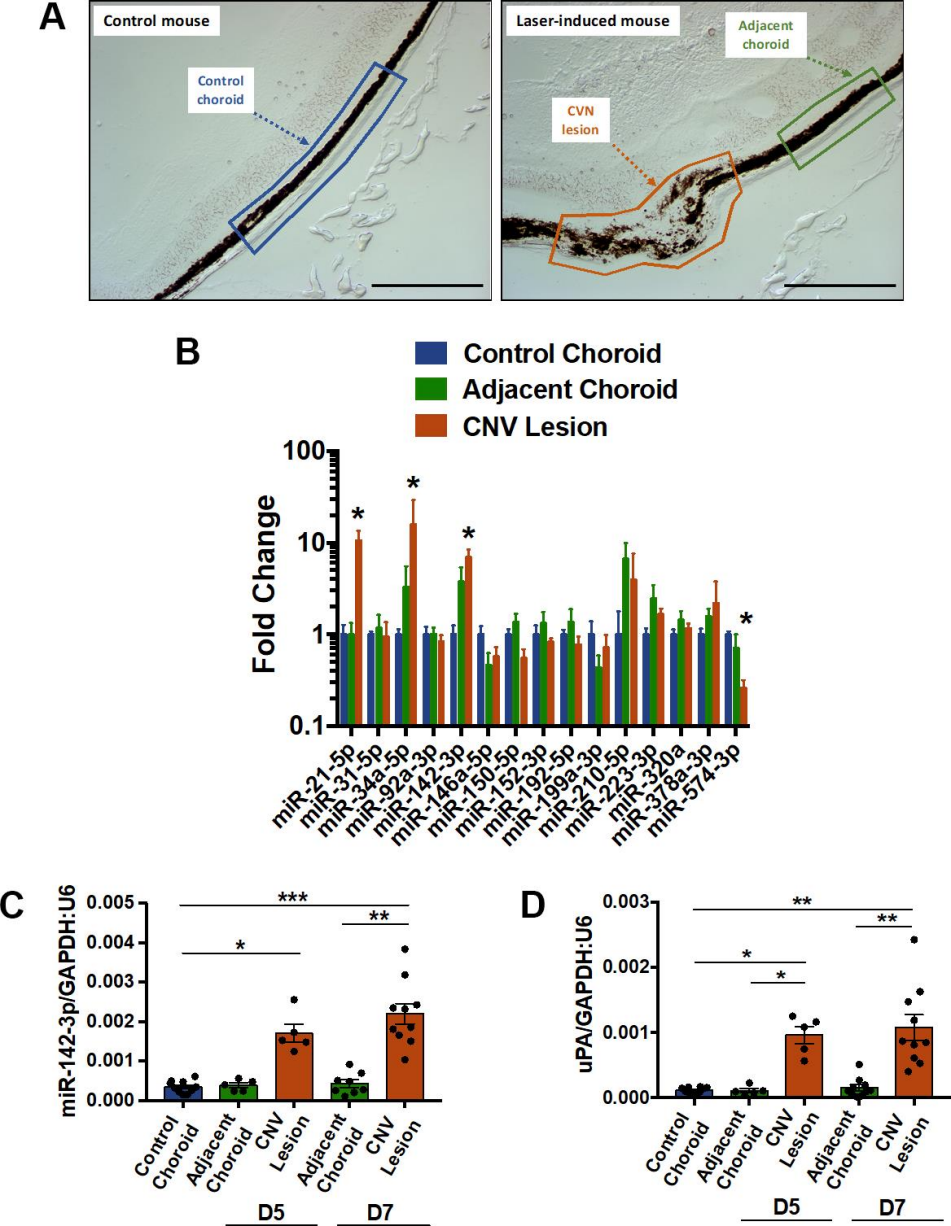
**MiR-142-3p is overexpressed in a laser-induced CNV mouse model.** (**A**) Cryosections of control mouse (untreated) and laser-induced mouse delineating the different regions of interest (ROI). These ROIs, namely control choroid (in blue), adjacent choroid (in green) and CNV lesion (in orange), were isolated by laser microdissection. Scale bar = 250 μm. (**B**) MicroRNA expression in CNV lesion and adjacent choroid compared to control choroid, 7 days after laser induction. Results of CNV lesion and adjacent choroid are expressed as fold change to control choroid. Results are presented as mean +- SEM. One-way ANOVA followed by multiple comparisons test (n = 5 per experimental group). (**C**, **D**) Overexpression of miR-142-3p (C), and uPA (D), a CNV-disease marker, at day 5 (D5) and day 7 (D7). MiR-142-3p and uPA are specifically overexpressed in CNV lesion 5 and 7 days post laser induction. qRT-PCR results are presented as mean +- SEM. One-way ANOVA + multiple comparisons test (n = 10 for control choroid, n = 5 for adjacent choroid and CNV lesion D5, n = 10 for adjacent choroid and CNV lesion D7) (* = p ≤ 0.05; ** = p ≤ 0.01; *** = p ≤ 0.001).

Laser microdissection was used to isolate three types of samples from mice subjected or not to laser-induced CNV: i) control choroid from control mice (not subjected to CNV induction), ii) CNV lesions, and iii) adjacent choroid (healthy choroid adjacent to CNV lesion) (Figure 1A). In a pilot experiment, the expression profile of 15 angiogenesis and/or inflammation-related microRNAs were determined 7 days post-induction by qRT-PCR in the 3 regions of interest (Figure 1B). Although probably not extensive enough, we focused our attention only on these 15 microRNAs i) because of limited RNA availability after laser microdissection and ii) because these microRNAs are reported angiogenesis and/or inflammation mediators. Among those microRNAs tested, only 4 were dysregulated: miR-21-5p, miR-34a-5p and miR-142-3p increased whereas miR-574-3p decreased upon laser-induction. To further confirm the overexpression of miR-142-3p in laser-induced CNV mouse, laser microdissection was repeated 7 days as well as 5 days post laser-induction. Interestingly, the miR-142-3p increased in the CNV lesions, both at days 5 and 7 post laser induction (Figure 1C), and correlated with the overexpression of uPA (urokinase plasminogen activator) used here as a CNV-disease marker [21] (Figure 1D and Supplementary Figure 1). Taken together, these data suggest that miR-142-3p could be of CNV progression in the laser-induced CNV mouse model.

### Modulation of miR-142-3p expression alters neovascularization and inflammation in CNV mouse model

**Figure 2:**
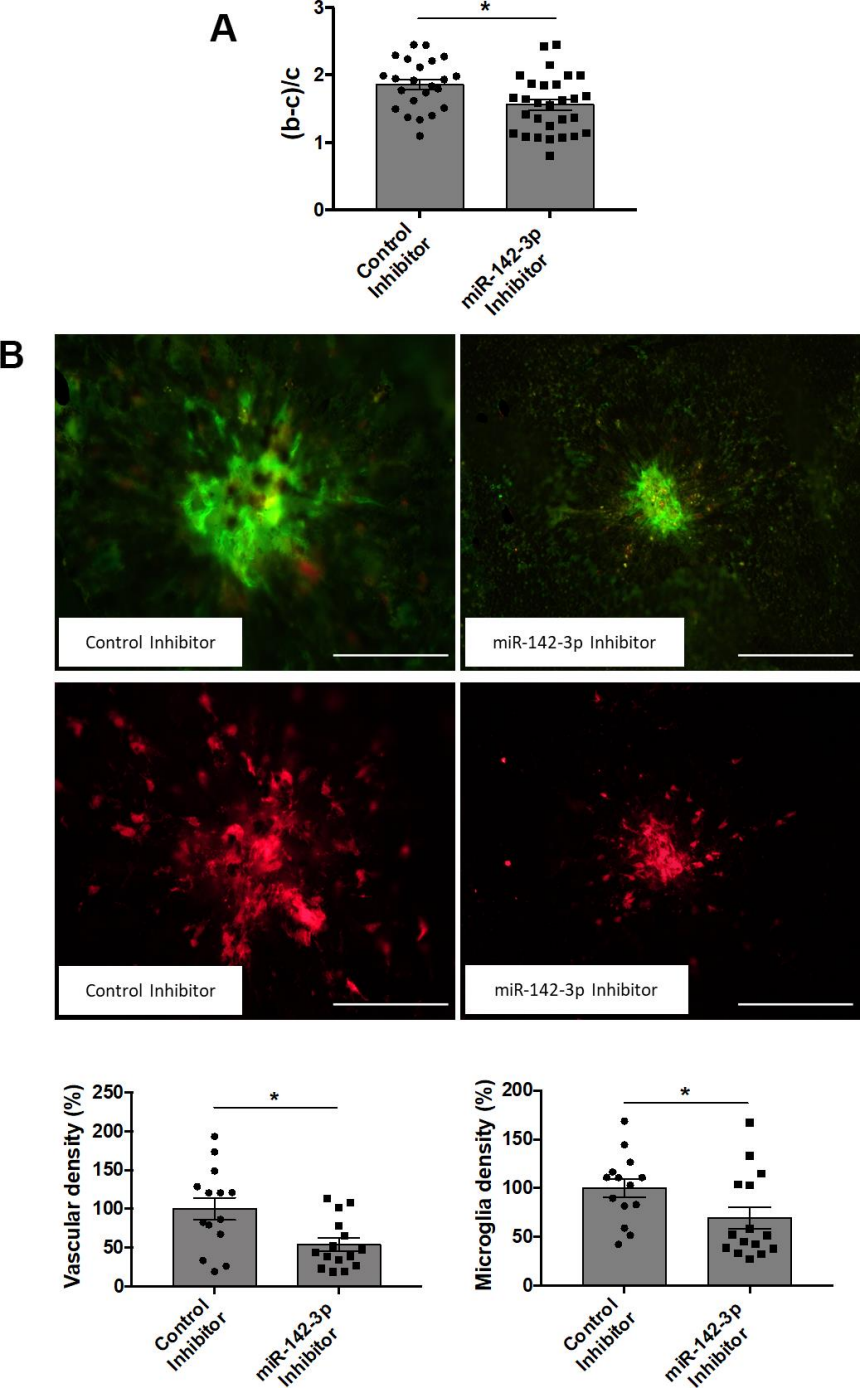
**Inhibition of miR-142-3p in a laser-induced CNV mouse model decreases both vascular and inflammatory phenotypes.** (**A**) OCT measurement presented as (b-c)/c ratios where b is the CNV lesion thickness and c is the adjacent choroid thickness. b and c were measured just prior to sacrifice (n = 23-29 per experimental group). (**B**) Flat-mounted choroids showing vascular (in green) and microglia (in red) density and corresponding quantification. Scale bar = 250 μm (n = 14-15 per experimental group). All results are presented as mean +- SEM. Mann Whitney test (* = p ≤ 0.05).

**Figure 3:**
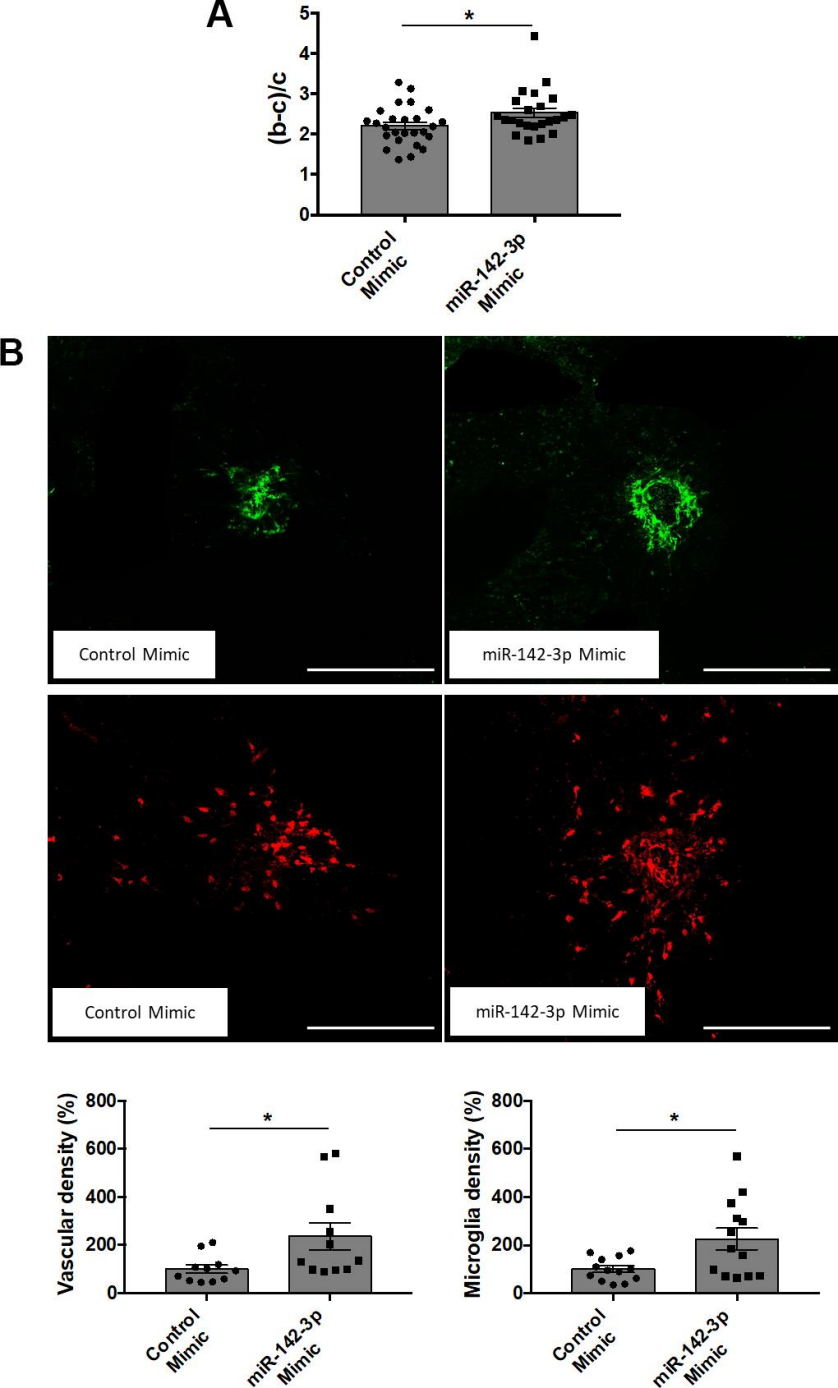
**Overexpression of miR-142-3p in a laser-induced CNV mouse model increases both vascular and inflammatory phenotypes.** (**A**) OCT measurement presented as (b-c)/c ratios where b is the CNV lesion thickness and c is the adjacent choroid thickness. b and c were measured just prior to sacrifice (n = 24-26 per experimental group). (**B**) Flat-mounted choroids showing vascular (in green) and microglia (in red) density and corresponding quantification. Scale bar = 250 μm (n = 11-13 per experimental group). All results are presented as mean +- SEM. Mann Whitney test (* = p ≤ 0.05).

Intravitreal injections of miR-142-3p inhibitor or mimic allowed us to evaluate the functional role of miR-142-3p during CNV progression in the laser-induced CNV mouse model. CNV lesion thickness, as assessed by OCT measurements, was lower in mice injected with miR-142-3p inhibitor when compared to control inhibitor (Figure 2A) (p = 0.0149). This inhibitor reduced by 46 % the blood vessel density in CNV mouse (Figure 2B, top panels) (p = 0.0157). Concomitantly, microglia area, a major component of the innate immunity in the eye, was decreased by 30 % upon miR-142-3p inhibition (Figure 2B, bottom panels) (p = 0.0292). Therefore, miR-142-3p inhibition alleviates CNV progression by decreasing neovascularization and inflammation. Inversely, the overexpression of miR-142-3p through mimic injection slightly enhanced CNV lesion thickness (Figure 3A) (p = 0.407). It also increased the angiogenic response by 136 % (Figure 3B, top panels) (p = 0.0233) and microglia cell recruitment by 126 % (Figure 3B, bottom panels) (p = 0.0441). For both inhibitor and mimic conditions, miR-142-3p retinal levels were measured to assess its inhibition/overexpression (Supplementary Figures 2, 3). Altogether, the modulation of miR-142-3p expression in CNV mouse model revealed a functional role of this microRNA during CNV progression. Intriguingly, both the vascular and microglia components of CNV lesions were affected by miR-142-3p modulation.

### Retina microglia cell activation is closely linked to miR-142-3p expression

**Figure 4:**
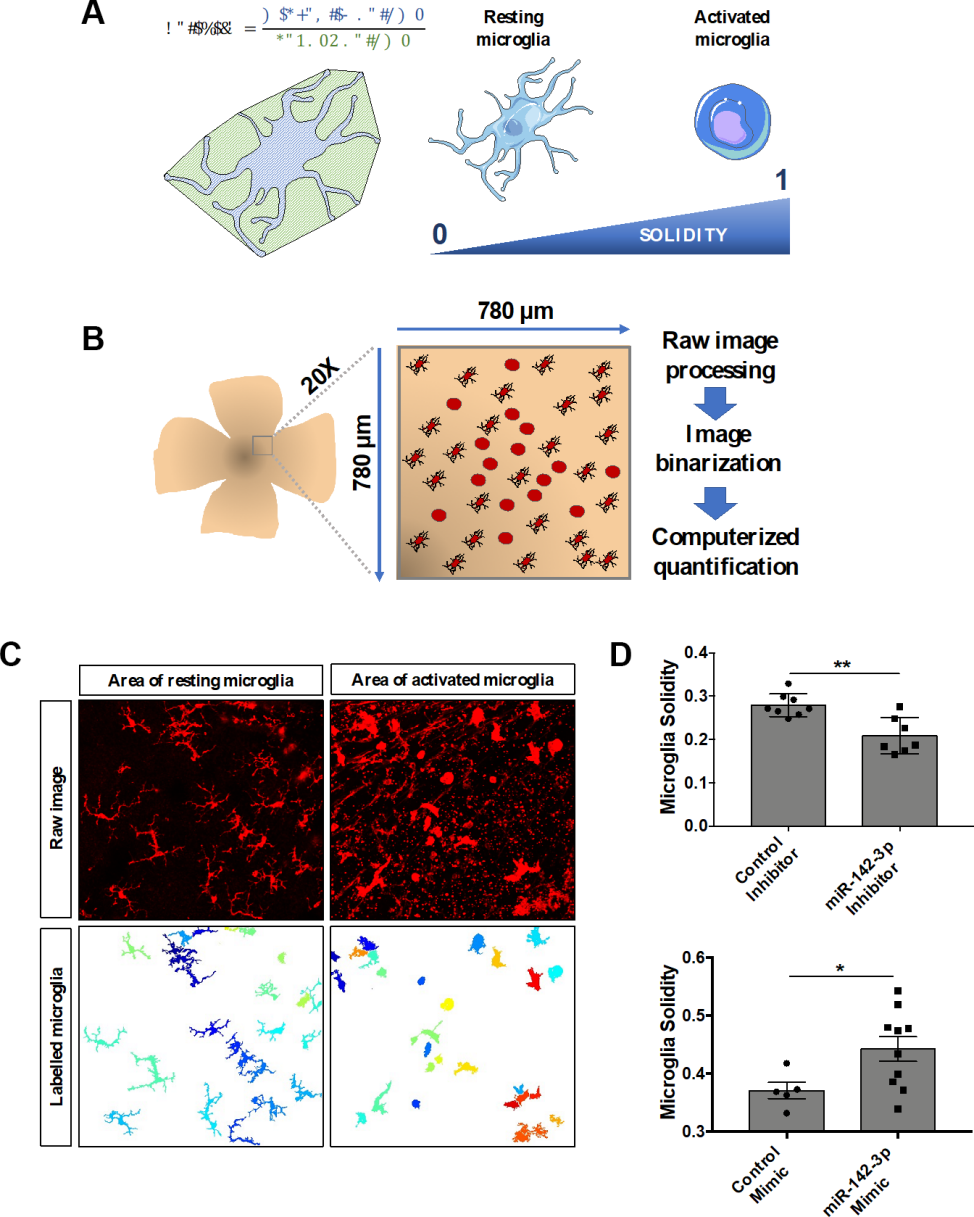
**MiR-142-3p influences microglia cell activation state *in vivo*.** (**A**) Characterization of microglia morphology via cell solidity. The solidity of an object is defined as the ratio between its volume and its convex volume. Resting microglia are highly ramified while activated microglia present an amoeboid shape, with no or small ramifications. Activated microglia are characterized by a higher solidity. (**B**) CNV lesion area of flat-mounted retinas and surrounding healthy tissue were imaged at the 20X magnification and then processed and quantified. (**C**) Representative raw images and corresponding labelled images of resting and activated microglia area. (**D**) Microglia activity measured around the CNV lesion in mice injected with either miR-142-3p inhibitor or mimic and relative controls (n = 5-10 per experimental group). All results are presented as mean +- SEM. Mann Whitney test (* = p ≤ 0.05; ** = p ≤ 0.01).

Microglia cells are resident immune cells of the central nervous system, including the retina. In laser-induced mouse model of CNV, microglia are recruited from the retina and invade the underlying CNV lesion [22, 23]. Under activation in inflammatory conditions, resting microglia cells lose their highly ramified morphology and gain an amoeboid shape. To assess the effect of miR-142-3p on retina microglia cell shape *in vivo*, an innovative computerized quantification method has been set up. Cell solidity corresponds to the ratio between the cell volume and its convex volume, and reflects microglia activation (Figure 4A). While activated amoeboid microglia cells are characterized by a solidity closer to 1 (which characterizes a round cell), resting ramified microglia cells are characterized by a solidity closer to 0 (Figure 4A). Each portion of the whole retina corresponding to a CNV lesion and its surrounding tissue was imaged at the 20X magnification to insure an optimal resolution for cell solidity analysis (Figure 4B). Raw images were processed and binarized for computerized quantification, which allowed a clear discrimination of every single microglia cell within the entire image (Figure 4C and Supplementary Figure 4). Interestingly, this computerized image analysis revealed that miR-142-3p inhibitor and miR-142-3p mimic impacted drastically the morphology of retina microglia cell (Figure 4D and Supplementary Figure 5). While miR-142-3p inhibitor decreased microglia solidity and thus microglia activation, miR-142-3p mimic had the opposite effect. Altogether, these data suggest that miR-142-3p contributes *in vivo* to microglia cell activation.

### miR-142-3p regulates the activation of human microglia cells under pro-inflammatory conditions

**Figure 5:**
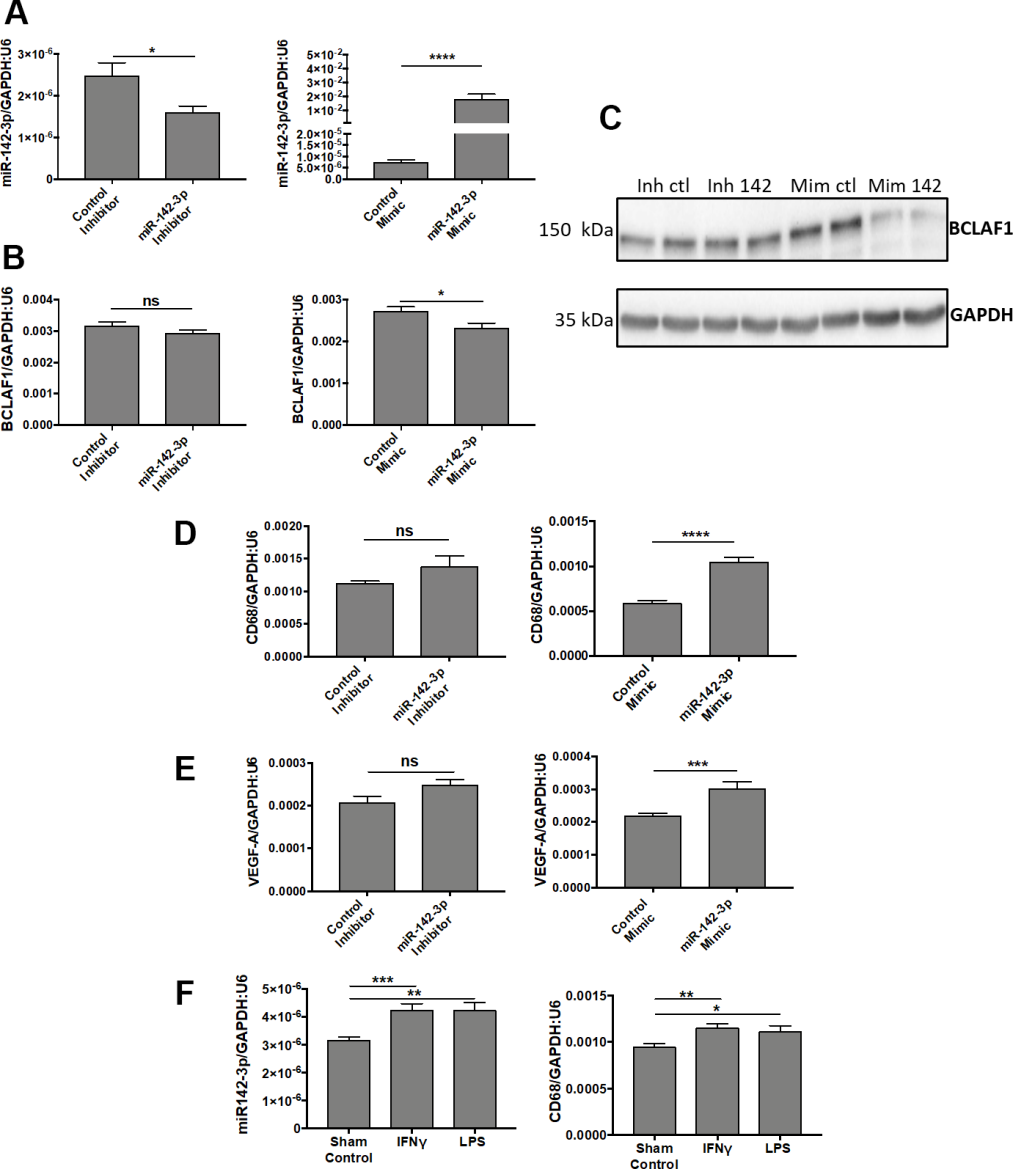
**MiR-142-3p regulates the activation of human microglia cells under pro-inflammatory conditions.** (**A**–**C**) Transfections of miR-142-3p inhibitor and mimic lead to decreased and increased miR-142-3p level, respectively (**A**). Transfection of miR-142-3p mimic decreased BCLAF1 levels, a previously identified target of miR-142-3p, both at the transcript level (**B**) and protein level (**C**). (**D**, **E**) Effects on CD68 level (**D**) or VEGF-A (**E**) in HMC3 transfected with miR-142-3p inhibitor or mimic and relative controls. Only miR-142-3p mimic was able to induce CD68 et VEGF-A production. (**F**) HMC3 stimulated with either IFN**ᵞ** or LPS for 24 hours overexpress miR-142-3p as long with CD68. All qRT-PCR results are presented as mean +- SEM. Mann Whitney test. (* = p ≤ 0.05; ** = p ≤ 0.01; *** = p ≤ 0.001; **** = p ≤ 0.0001; ns = not significant).

Transfection of human microglia HMC3 cells with miR-142-3p mimic drastically increased miR-142-3p expression (Figure 5A, right panel). It also decreased the production of its previously described target, BCLAF1, both at mRNA (Figure 5B, right panel) and protein (Figure 5C) levels [24]. BCLAF1 was used here to validate the efficacy of miR-142-3p mimic. Alongside with its effect on BCLAF1 expression, miR-142-3p overexpression also influenced HMC3 activation state as assessed by increased CD68 level (Figure 5D, right panel), a marker of microglia activation [25, 26]. Interestingly, VEGF-A transcript level (Figure 5E, right panel) increased upon miR-142-3p mimic, which could contribute to the effect observed *in vivo* on the angiogenic response. When using the inhibitor, only a slight decrease of miR-142-3p expression was observed (Figure 5A, left panel) without any effect on BCLAF1, CD68 and VEGF-A levels (Figure 5B–5E, left panels). Such a low effect of the inhibitor may be related to the low basal level of miR-142-3p in HMC3 cell line.

Our data *in vitro* confirm the *in vivo* findings of the biological implication of miR-142-3p in microglia activation. This is further supported by the concomitant increase of miR-142-3p and CD68 expressions in HMC3 cells stimulated by IFNᵞ or LPS (Figure 5F). Taken together, these data revealed a strong link between the activation state of microglia cell and miR-142-3p expression.

## DISCUSSION

The present study unveils a functional role of miR-142-3p in stimulating CNV formation in a mouse model of wet AMD. This novel concept is supported by i) the overexpression of miR-142-3p in CNV lesions, and by ii) the impact of this miRNA modulation (inhibition/overexpression) on both vascular and inflammatory phenotypes. While inhibition of miR-142-3p caused a subsequent decrease of angiogenesis, its overexpression had the opposite effect. Beside its effect on angiogenesis, administration of miR-142-3p activates microglia cells -part of the inflammatory process- both *in vivo* and *in vitro*. Our data provide evidence for a pivotal role played by miR-142-3p during CNV progression and particularly on microglia activation.

The originality of our approach relies on the laser microdissection, which enables us to isolate CNV tissue devoid of any surrounding healthy tissue that could have biased the microRNA profile [27]. Indeed, previous studies in CNV mouse primarily investigated microRNAs circulating in the blood [17, 28] or present in the entire RPE-choroid-sclera complexes [29]. It is noteworthy that CNV lesion sites only englobe 2.5% of total choroid tissue, making entire choroid complexes highly heterogenous mixes of healthy and non-healthy tissue. Among the microRNAs tested, 4 were dysregulated: miR-21-5p, miR-34a-5p and miR-142-3p increased, whereas miR-574-3p decreased upon laser-induction. In line with our data, miR-21-5p was previously reported as strongly up-regulated in RPE-choroid-sclera complex [29]. MiR-21-5p is a well-known pro-angiogenic microRNA in different pathologies [30, 31] and is highly abundant in human retinal endothelial cells [32]. Concomitantly to the overexpression of miR-34a-5p in our samples, this microRNA was found to be upregulated in serum of AMD patients [33] and in hydrogen peroxide-induced prematurely senescent ARPE-19 cells [34], possibly leading thus to AMD progression. Concerning miR-574-3p, its downregulation in the CNV model did not prompt us to consider it for an inhibition strategy.

Here, we identified miR-142-3p as a specific CNV lesion microRNA, being upregulated both 5- and 7-days post laser-induction and displaying a correlation with uPA, a well-established CNV disease marker [21]. MiR-142 gene is a broadly conserved miRNA gene among species that gives rise to two distinct mature forms, miR-142-3p and miR-142-5p. MiR-142 is involved in stem cell fate, cardiomyocyte hypertrophy, cancer, immune tolerance and hematopoiesis, as previously reviewed [35]. Both vascular [24] and inflammatory [36] functions have been attributed to this microRNA. Interestingly, miR-142-3p expression is upregulated in a mouse model of retinal degeneration [37], but its biological role in ocular disease, including wet AMD, remained unknown. The increase of miR-142-3p in CNV goes in parallel with macrophage recruitment, as well as the increase in vascular density [7]. Thus, miR-142-3p expression was associated with two interconnected hallmarks of the disease, namely inflammation and angiogenesis. Interestingly, miR-142-5p, the other mature strand of miR-142, was not detected in our laser-induced CNV mouse model. This lack of expression may reinforce the specific function of miR-142-3p only, and not miR-142-5p, during CNV progression. Previous studies revealed that the cellular source of miR-142-3p may be more immune-related rather than vascular-related [38–40]. Accordingly, miR-142-3p expression in human microglia cells increased upon IFNᵞ or LPS stimulation, and correlated with CD68 level, a marker of activated microglia. To evaluate the functional role of miR-142-3p during CNV, miR-142-3p inhibitor or mimic were injected into the vitreous chamber of CNV mouse. Intravitreal injections are routinely used by clinicians to deliver anti-VEGF drugs to wet AMD patients [41]. On one hand, intravitreal injection of miR-142-3p inhibitor decreased the vascular density in flat-mounted choroids, while injection of miR-142-3p mimic conversely increased it. Those findings were further confirmed by alterations of CNV lesion thickness measured via OCT. Furthermore, intravitreal miR-142-3p inhibition alleviated microglia density, while miR-142-3p mimic increased it. The biodistribution of miR-142-3p inhibitor/mimic within mouse eyeball after intravitreal injections is not known. Still, we noticed an impact on both retinal and choroidal tissues, suggesting a wide diffusion of the molecule. Before using this kind of inhibitor in the clinic, a better understanding of its diffusion within the eye and a target cell optimization would be needed. These improvements may imply less frequent injections than current therapy.

Microglia are the immune resident cells of the central nervous system, constantly screening their environment for pathogens and ready to migrate, proliferate and phagocyte if needed. In physiological conditions, retinal microglia cells exhibit a highly ramified morphology, shifting to a more amoeboid shape under activation [23]. In the laser-induced CNV context, microglia are known to migrate from retina and invade CNV lesion site, starting 4-days post laser-induction [22]. Various cellular morphology parameters exist to describe microglia shape [42, 43], but cell solidity has been described as the most appropriate one for assessing the amoeboid shape of activated microglia, and thus microglia activation [44]. An innovative computerized imaging and quantification technique revealed that miR-142-3p inhibition/overexpression decreases/increases retinal microglia cell solidity *in vivo*, in line with our *in vitro* data of miR-142-3p mediated microglia activation. Our data suggest that miR-142-3p stimulates the activation of microglia in the retina and their migration to the CNV lesion site, located within the choroid. Accordingly, miR-142-3p is one of the top up-regulated microRNA in primary microglia isolated from rat pup’s brain under LPS stimulation [45]. Besides morphological analysis, future studies may evaluate mouse microglia activation state through transcriptomic and surface marker analyses. Interestingly, in human microglia culture, miR-142-3p mimic enhanced drastically miR-142-3p levels, as well as CD68 expression, a marker of activated microglia [25, 26]. An interesting finding is the concomitant up-regulation of VEGF-A, a key molecular mediator of CNV progression [46]. This demonstrates that miR-142-3p is able to modulate both inflammatory and vascular responses, two intimately linked processes associated to CNV progression [47]. Interestingly, CD68 was also upregulated in HMC3 stimulated by IFNᵞ or LPS, leading to a pro-inflammatory state [48, 49]. This observation supports the concept that miR-142-3p is a mediator of inflammation triggering microglia cell activation and is in line with our *in vivo* data. How miR-142-3p modulates CD68 and VEGF-A production remains to be elucidated. However, our data support the concept that miR-142-3p could be a new intermediate in the vascular-inflammatory axis during CNV progression, through microglia activation.

In conclusion, miR-142-3p may exacerbate CNV by enhancing microglia migration and activation, and therefore is worth considering as a potential therapeutic target in AMD.

## MATERIALS AND METHODS

### Laser-induced choroidal neovascularization mouse model

CNV was induced in mice by laser-mediated choroid burn as previously described [7]. Briefly, 6 to 8 weeks old C57Bl6 mice (all males) (Janvier, France) were anesthetized by intraperitoneal injection of a mixture of ketamine (50 mg/kg) and medetomidine (0.5 mg/kg). A few minutes before laser induction, pupils were dilated with Tropicol (Théa Pharma, France). Four laser burns per eye were performed with MicronIV instrument (Phoenix Lab, USA) leading to Bruch’s membrane local destruction and the subsequent choroidal neovascularization. Mice were than woken up by intraperitoneal administration of atipamezole (1mg/kg). Mice were sacrificed 5 (D5) or 7 days (D7) after laser induction by cervical dislocation. All *in vivo* experiments were approved by the local Animal Ethic Commission (File number 17-1986, “Commission d’éthique de l’utilisation des animaux de l’Université de Liège”).

### Laser microdissection of the choroid

Mice subjected or not to CNV induction were sacrificed at D5 or D7 post-laser burn (n = 5-10 per experimental group, 4 CNV lesions per eye). Eyes were enucleated, placed fresh in Tissue-Tek OCT medium (VWR, USA) and stored at -80° C until section preparation and laser microdissection. The day of laser microdissection, eyes were placed on dry ice until cryosection (Cryostat Cryotome FSE, Thermo Scientific). Ten μm sections were performed and place on PEN-Frame slides (Leica, Germany). Regions of interest (ROIs) were microdissected with Leica Laser Microdissection LMD 7000 and directly collected in lysis buffer for subsequent RNA extraction. Samples were collected from 3 different ROIs: control choroid (from control mouse not subjected to CNV), CNV lesion (neovascular tissue from laser-induced mouse) and their corresponding adjacent choroid (healthy tissue next to CNV lesion from laser-induced mouse). For each ROI, microdissected tissues from the two eyes of a single mouse were pooled before RNA extraction.

### Intravitreal injections and optical coherence tomography (OCT)

To assess inhibition/overexpression of miR-142-3p, intravitreal injections of inhibitor (single stranded LNA-antimiR) or mimic (chemically modified double-stranded) were performed in mice, just after laser induction (D0, inhibitor conditions), or just after laser induction and 5 days later (D0 + D5, mimic conditions) (n = 11-15 per experimental group for flat-mounted choroids; n = 5-10 per experimental group for flat-mounted retinas). During general anesthesia, mouse eyes were locally anesthetized with Unicaïn 0.4% (Théa Pharma) before the injection, in the vitreous chamber, of a solution (2 μL) of inhibitor (1.83 mM) or mimic (10 μM). Negative control inhibitor and miR-142-3p inhibitor were purchased from Qiagen (miRCURY LNA miRNA Inhibitors, The Netherlands). Mimics (negative control and miR-142-3p mimic) were from Ambion (mirVana miRNA Mimics, USA). LNA-antimiR-142-3p sequence is 5’-AAGTAGGAAACACTAC-3’. After intravitreal injection, eyes were covered with Trafloxal (Bausch + Lomb, Austria) to avoid any infection and inflammation. Both eyes of a single mouse received the same treatment. Phenotypic alterations consequent to miR-142-3p modulation were assessed through optical coherence tomography (OCT) measurements by using the MicronIV instrument, through vascular and microglia stainings on flat-mounted choroids, and through microglia staining on flat-mounted retinas. OCT measurements are presented as (b-c)/c ratios, where b is the CNV lesion thickness and c the adjacent choroid thickness [7]. For miR-142-3p mimic conditions, FITC-dextran (FD2000S, Sigma, USA) tail vein injections were performed 3 minutes before sacrifice.

### RNA extraction, reverse transcription and qRT-PCR

Laser-captured choroids or cells were homogenized in lysis buffer by vortexing. For some qRT-PCR, whole retinas and choroids were lysed with ceramic beads (MagNA Lyser Green beads, Roche, Switzerland) in lysis buffer with tissue homogenizer (Precellys Evolution, Bertin, France). RNA was then extracted with miRNeasy Micro kit (laser-captured choroids) (Qiagen) or with mirVana kit (all other experiments) (Ambion), according to the manufacturer’s protocols. Complementary DNA was synthesized with miScript RT Kit II (Qiagen) using the same RNA input according to the manufacturer’s instructions. mRNA levels of different targets and a set of microRNA levels were quantified by qRT-PCR using FastStart SYBR Green Master (Roche) and a LightCycler 96 instrument (Roche). MiRNA levels were assessed with miRCURY LNA miRNA PCR Assay (Qiagen). All data were normalized to GAPDH and snU6 expression as housekeeping genes. Primer sequences used to detect mouse and human target mRNAs are presented in Table 1. The qRT-PCR results are presented as expression ratios between gene (or microRNA) of interest (GOI) expression and normalizers (NOR) expression. The methodology used for normalization was the following:

$$\frac{2^{-Ctmean\;GOI}}{2^{-Ctmean\;NOR}}=GOI\;expression\;ratio$$

**Table 1:** Forward (f) and reverse (r) primer sequences for mouse (mmu) and human (hsa) target mRNAs.

	**5’ - sequence - 3’**
**mmu-GAPDH**	f: ggtggacctcatggcctaca
	r: ctctcttgctcagtgtccttgct
**mmu-uPA**	f: taaaatgctgtgtgctgcgg
	r: gcggccttcgatgttacaga
**mmu-hsa-snU6**	f: cgcttcggcagcacatatac
	r: ttcacgaatttgcgtgtcat
**hsa-GAPDH**	f: acccactcctccacctttgac
	r: accctgttgctgtagccaaatt
**hsa-CD68**	f: cagggaatgactgtcctcaca
	r: ctctgtaaccgtgggtgtca
**hsa-VEGFa**	f: cctccgaaaccatgaacttt
	r: atgattctgccctcctctt

### Western blotting

Cells were lysed with 1X lysis buffer (9803, Cell Signaling, USA) containing protease and phosphatase inhibitors (Complete and Phos-STOP, Roche). Proteins (15 μg) were separated on acrylamide gels and transferred onto PVDF membranes. BCLAF1 proteins were detected by overnight incubation, at 4° C, with recombinant anti-BTF antibody (1/10 000 dilution) (Ab181240, Abcam, UK) followed by 1h incubation, at room temperature, with horseradish peroxidase-coupled secondary antibody (7074, Cell signaling) and enhanced chemiluminescent substrate (NEL1040001EA, PerkinElmer, USA) using a LAS4000 imager (Fujifilm). GAPDH (1/10 000, MAB 374, Millipore, USA) was used as a loading control.

### Immunohistochemistry

For mouse microglia immunostaining, eyes were enucleated and fixed in paraformaldehyde 4% for 1 hour. Subsequently, retinas and choroids were isolated, permeabilized, blocked and incubated with anti-Iba1 antibody (1/1000 dilution) (Abcam Ab178846) overnight, at room temperature. The next day, samples were washed, incubated with secondary antibody (1/200 dilution) (goat anti-rabbit AlexaFluor 595, Invitrogen A11012, USA) for 2 hours and washed again. Retinas and choroids were flat-mounted with Fluoromount-G (SouthernBiotech, The Netherlands) on glass-slides for microscopy imaging.

For mouse vascular immunostaining, eyes were enucleated and fixed in ethanol 70 % for 1 hour. Subsequently, choroids were isolated, permeabilized, blocked and incubated with anti-CD31 antibody (1/150 dilution) (Pharmingen 553370) overnight, at room temperature. The next day, samples were washed, incubated with secondary antibody (1/200 dilution) (goat anti-rat AlexaFluor 488, Invitrogen A11006) for 2 hours and washed again. Choroids were flat-mounted with Fluoromount-G (SouthernBiotech) on glass-slides for microscopy imaging.

### Epifluorescence/confocal microscopy and image quantifications

Flat-mounted Iba1/CD31 stained choroids were observed under an Olympus Vanox epifluorescence microscope (inhibitors conditions). Flat-mounted Iba1/FITC stained choroids were observed under a Leica Sp5 confocal microscope (mimics conditions) and Z-stacks (z-path = 2.5 μm) were taken. Each CNV lesion was separately imaged and maximum intensity projections were performed for Iba1/FITC images. For each CNV lesion, the area occupied by the staining was calculated with Fiji software [50]. The mean area for a single eye was than calculated for each staining. The percentage of stained area within an eye, normalized to control condition, was used for subsequent statistical analysis to determine vascular and microglia densities.

For cell solidity analysis, Z-stacks of flat-mounted Iba1 stained retinas were taken with a high-resolution confocal microscope (Zeiss LSM 880 with Airyscan), at the 20X magnification. Every CNV lesion was separately imaged, each image representing a 780 μm long square, englobing one CNV area and the surrounding tissue. After acquisition, images were first processed and then quantified. The processing procedure started by identifying image background with morphological opening operations and subtracting it from the raw images. They were then thresholded using Otsu’s method and subjected to a median filter to reduce noise. First quantification steps consisted in detecting every objects of the image and discarding the ones which were too small/big to be an isolated cell or a small cell cluster. The bright center of the cells was then used to segment and isolate each cell. Remaining non-cellular objects were discarded using shape and size filters. Finally, tridimensional solidity was measured for every cell. The mean cell solidity of all the CNV lesions area of a single eye was calculated and then used for subsequent data analysis. The solidity of an object is defined as the ratio of the volume of this object over its convex volume. The convex volume corresponds to the volume of the convex hull of the region, i.e. the smallest region that satisfy two conditions: (1) it is convex (2) it contains the original region.

### Cell culture

Human microglia HMC3 cells were purchased from ATCC (CRL-3304, USA) and cultured according to the manufacturer’s instructions. Cells were seeded in 6-well plate (2.10^5^ cells per well) the day before transfection or stimulation. HMC3 were transfected with 5 nM of miR-142-3p inhibitor/negative control inhibitor or with 5 nM miR-142-3p mimic/negative control mimic. Transfection was performed using INTERFERin reagent (Polyplus transfection) following manufacturer’s protocol. Cells were harvested 24-hour post-transfection to assess miR-142-3p, BCLAF1 transcript and VEGF-A levels, or 48-hour and 72-hour to assess CD68 and BCLAF1 protein levels, respectively. Alternatively, HMC3 underwent pro-inflammatory stimulation by a 24 hours exposure to 10 ng/mL IFNᵞ (Recombinant Human IFNᵞ, Peprotech, USA) or to 0,1 μg/mL LPS (Lipopolysaccharides from *Escherichia coli* 0111:B4, Sigma, USA).

### Data analysis

All data and measurements underwent a statistical analysis using Prims7 software. All graphs are presented as mean +/- SEM. Mann-Whitney test or Kruskal-Wallis followed by post-hoc multiple comparisons tests were used to determine statistical significance. Statistical significance was defined as p ≤ 0.05.

### Ethics approval

All *in vivo* experiments were approved by the local Animal Ethic Commission (File number 17-1986, “Commission d’éthique de l’utilisation des animaux de l’Université de Liège”).

### Availability of data and material

All data generated or analyzed during this study are included in this manuscript and its figures.

### Code availability

Custom code for microglia solidity analysis is available on demand.

## Supplementary Material

Supplementary Figures
