# Supplementary material for: Intravitreal injection of anti-miRs against miR-142-3p reduces angiogenesis and microglia activation in a mouse model of laser-induced choroidal neovascularization
Source: Aging (Albany NY). 2021 May 5;13(9):12359–77. doi: 10.18632/aging.203035 (PMC8148470; doi:10.18632/aging.203035)
Supplement: Supplementary Figures [file aging-13-203035-s001.pdf]

SUPPLEMENTARY FIGURES

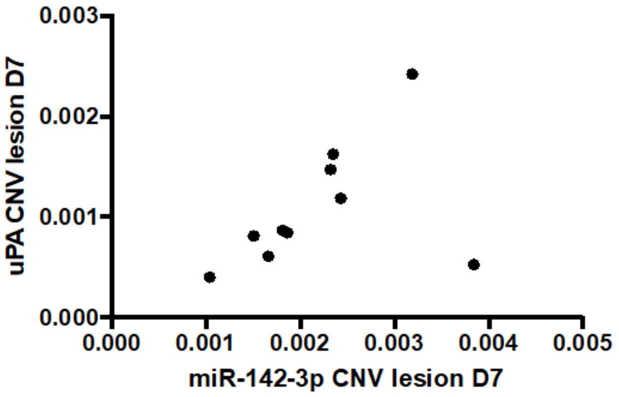

Supplementary Figure 1. Spearman correlation between uPA level and miR-142-3p level in CNV lesion 7 days post laser induction (r = 0.503; p = 0.144).

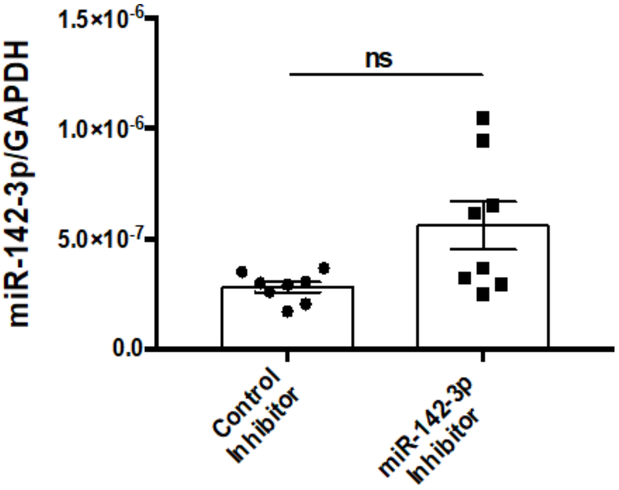

Supplementary Figure 2. Expression of miR-142-3p was measured in CNV mouse retinas 7 days post laser-induction and intravitreal injection of miR-142-3p inhibitor and relative negative control (n = 8 per experimental group) (ns = not significant).

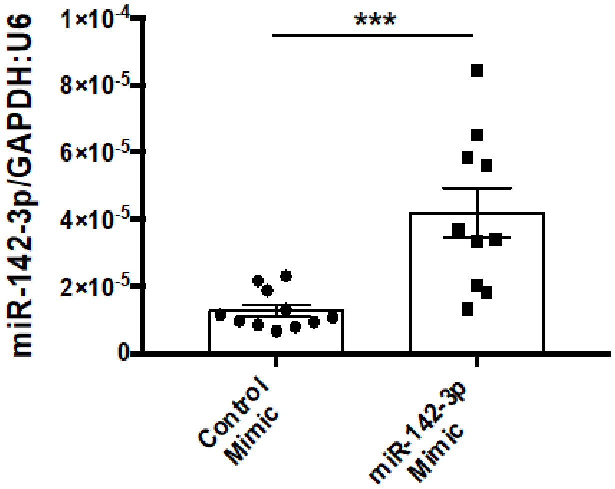

Supplementary Figure 3. Expression of miR-142-3p was measured in CNV mouse retinas 7 days post laser-induction and intravitreal injection of miR-142-3p mimic and relative negative control (n = 10-11 per experimental group) (* = p ≤ 0.001).**

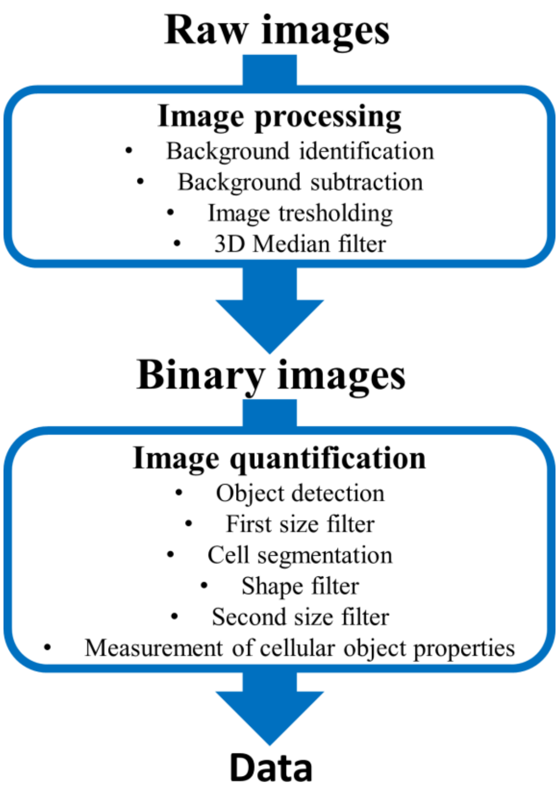

Supplementary Figure 4. Workflow chart of image processing and quantification for microglia solidity analysis in flat-mounted retinas.

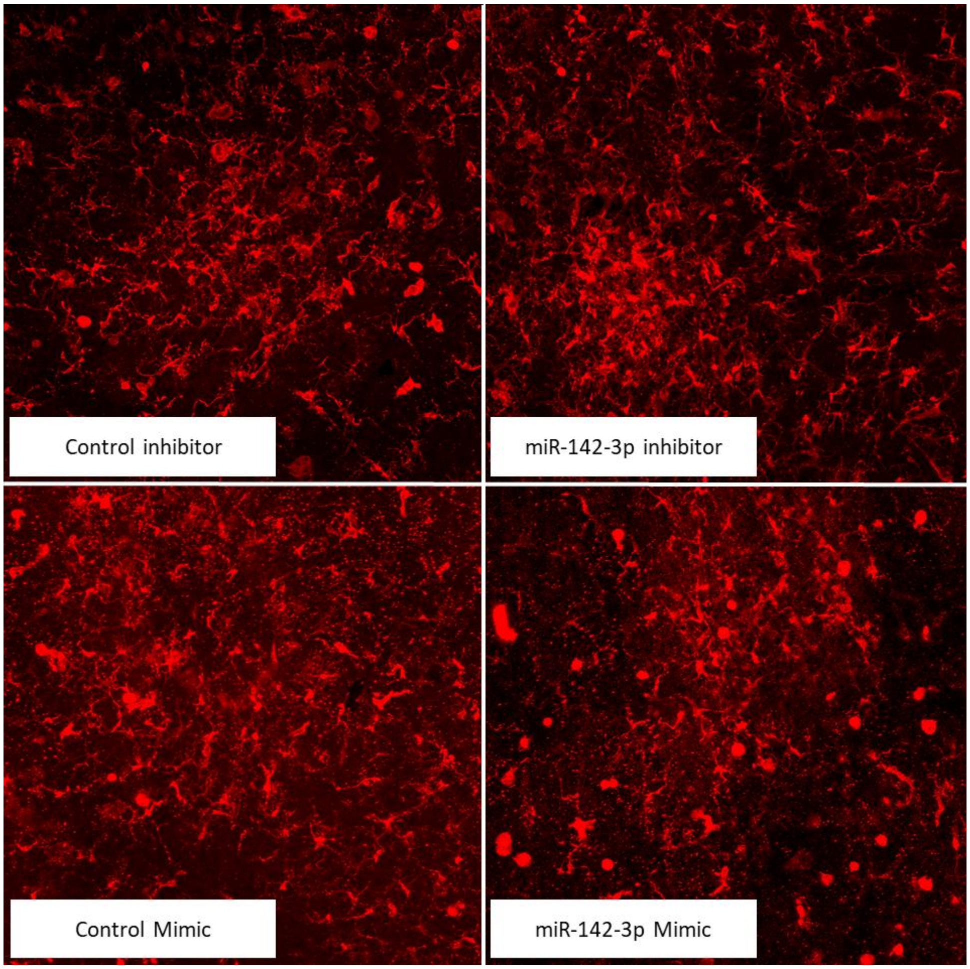

Supplementary Figure 5. Representative raw images of Iba-1 stained flat-mounted retinas for miR-142-3p and relative control inhibitors (top panels) and miR-142-3p and relative control mimics (bottom panels) treatment.
